# Editorial Research Topic: Non-coding RNA as Therapeutic Target: A Game Changer in Cardiac Regenerative Strategies?

**DOI:** 10.3389/fphys.2021.822280

**Published:** 2022-01-03

**Authors:** Julia Leonardy, Christian Bär

**Affiliations:** ^1^Hannover Medical School, Institute of Molecular and Translational Therapeutic Strategies, Hannover, Germany; ^2^REBIRTH Center for Translational Regenerative Medicine, Hannover Medical School, Hannover, Germany; ^3^Fraunhofer Institute for Toxicology and Experimental Medicine, Hannover, Germany

**Keywords:** non-coding RNA, microRNAs, lncRNAs, cardiac regeneration and development, cardiovascular disease, circRNA

## Introduction

Cardiovascular diseases are the most common cause of death worldwide (Roth et al., 2017). Though recent advances in therapeutic drug treatments made it possible to better manage the disease and increase the life span of heart failure patients, there is still no option to cure the patient's heart after a myocardial infarction (MI). This results mainly from a lack of cardiac regeneration after massive cardiomyocyte loss due to MI. Adult cardiomyocytes are mostly inable to divide. Their proliferation capacity gets lost after birth when maturation takes place and the cardiomyocyte become quiescent (Tzahor and Poss, 2017). Recent research is focused on deciphering of the molecular switches from neonatal to adult cardiomyocytes, i.e., regenerative vs. post-mitotic states of cardiomyocytes.

Non-coding RNAs (ncRNAs) account for the vast majority of mammalian transcripts and it has been shown in the last two decades that this class of molecules is involved in the regulation of many, if not all, physiological and pathological settings, including cardiac disease and regeneration (Bär et al., 2016; Beermann et al., 2016; Hunkler et al., 2021).

Due to their involvement in many different pathological settings, ncRNAs serve as attractive therapeutic targets. Synthetic ncRNA mimics can be used to restore decreased function, whereas anti-sense oligonucleotides can be used to inhibit disease-promoting functions. Several ncRNA-based drugs are already in clinical trials, including those to target pathological heart conditions (Huang et al., 2020).

This Frontiers Research Topic entitled “Non-coding RNA as Therapeutic Target: A Game Changer in Cardiac Regenerative Strategies?” has collected nine contributions from experts who showcase recent findings of different aspects of cardiac regeneration and provide novel insights into the major subtypes of ncRNAs, particularly, long non-coding RNA (lncRNA), microRNA (miRNA), and circular RNA (circRNA) biology and their regulation in cardiovascular disease.

## Featured Publications

In the last decade, the miRNA-family 212/132 was shown to be crucial for the development of cardiac remodeling after pressure overload. Transgenic overexpression in cardiomyocytes led to the development of cardiac hypertrophy, whereas miR-212/132-knockout mice were protected (Ucar et al., 2012). In this Research Topic, Lei et al. investigated the effect of miR-212/132 knockout in mice after myocardial infarction (MI). Under baseline conditions cardiac contractile function in miR-212/312 knockout mice was improved, whereas 1 day and 4 weeks after MI no significant difference was found. These findings are partially surprising as Ucar et al. (2012) observed no differences in cardiac functions between knockout (KO) and wildtype (WT) mice under basal conditions and several other reports highlight cardiac improvement after miR-212/132 knockout in MI (Foinquinos et al., 2020; Batkai et al., 2021). These contradictory findings could be due to timing issues. Moreover, in a recent publication Lei et al. (2021) show that in a mouse model of pressure overload miR-212/132 knockout leads to improved cardiac function in comparison to WT animals. Importantly, the underlying mechanisms seem to be conserved in mammals, as therapeutic targeting of the miR-212/132 family in mice and pigs showed promising results for the treatment of heart failure (Foinquinos et al., 2020; Batkai et al., 2021). Based on these data, the worldwide first miRNA-antisense therapy targeting miR-132 in patients with chronic heart failure was launched and showed encouraging results (Täubel et al., 2021), underlining the outstanding role of miR-132 in the cardiac remodeling process.

Atherosclerosis is a leading cause of death and disability. Recent research showed that abnormal proliferation of vascular smooth muscle cells (VSMC) has a critical role in the formation of atherosclerotic lesions. One therapeutic option is to target mitogenic-induced proliferation of VSMCs. Tian et al. addressed this question by generating an expression profile of circRNAs in VSCMs treated with the mitogen PDGF-BB. They not only found 112 differentially expressed circRNAs, but also investigated the circRNA parental genes. Furthermore, Tian et al. provided bioinformatics-based analysis of a regulatory relationship between differentially regulated circRNA and VSCM-related miRNA paving the way for future therapeutic options.

Desjarlais et al. describe a RNA-sequencing approach to identify deregulated miRNAs in bone marrow-derived proangiogenic cell (PAC) after exposure to cardiovascular risk factors. PACs are important for postnatal neovascularization and thus play a crucial role in the handling of peripheral artery disease (Asahara et al., 1999). Interestingly, it became evident that several miRNAs were affected in all exposed conditions, indicating a general pattern to target for therapy.

A detailed and in-depth review and meta-analysis from Zhai et al. focused on studies from the last decade of miRNAs circulating in the blood after acute myocardial infarction (AMI). First, the authors summarize the findings from the selected publications. Next, the authors re-assessed the reported results. Even though they found that cardiomyocyte-specific miR-499 had better diagnostic accuracy than other single miRNAs, they still suggest to test a panel of several miRNAs rather than a single one in the future to serve as diagnostic markers for AMI.

Restricted blood flow in the heart due to blockage of the artery can lead to myocardial infarction and cardiac cell death. To overcome this problem, promoting cardiac neovascularization has been in the focus of recent research. In the review article of Kesidou et al. the process of neovascularization and the possibilities to target this process after MI is discussed. Recently, it has been shown that extracellular vesicles (EV) are important for cell-cell communication. Kesidou et al. describe current developments in targeting neovascularization using miRNAs and EV-bound miRNAs.

The review of Yuan and Krishnan summarizes the current knowledge on ncRNAs in cardiac regeneration and its potential to treat heart failure through the induction of cardiac regeneration. The role of miRNAs in cardiac differentiation, cardiomyocyte proliferation, cardiac reprogramming, and cardiomyocyte survival is described in detail, since this class of ncRNAs was the first discovered and many studies have been conducted since then. In addition, lncRNAs and circRNAs are described as important players in the field of cardiac regeneration, which becomes evident by the raising numbers of published manuscripts. Furthermore, the authors summarize recent attempts and successes of novel therapeutic strategies to treat heart failure through ncRNA-driven regeneration.

Santos et al. focus specifically on the role of lncRNA in cardiomyocyte proliferation and cardiac regeneration. The authors state, that molecular and physiological alterations during the aging of the heart facilitates the development of heart failure. This provides a potential role for lncRNAs as targets for therapeutic options. Furthermore, the possibility of direct reprogramming of endogenous cardiac fibroblasts to cardiomyocytes is discussed in order to replace damaged cells in the heart.

Another important aspect of lncRNAs and cardiac regeneration is the regulation of metabolic signaling. The switch from neonatal to adult cardiomyocytes is accompanied by a change of energetic source. The review of Correia et al. highlights findings from a possible molecular interplay of lncRNAs and metabolic signaling in regard to cardiac regeneration.

Complementing the other reviews in this Research Topic, the article of Mester-Tonczar et al. summarizes the function of circRNAs in cardiac regeneration. The authors give a comprehensive overview about circRNA biogenesis and their function in general. Later, they focus on their role in the setting of cardiomyocyte biology, cardiovascular diseases, and a possible therapeutic potential in this field. Mester-Tonczar et al. address in detail the implication of cirRNAs in MSC-derived and iPSC-derived cardiomyocytes and show the obstacles to cope with when studying circRNA biology.

## Summary

Non-coding RNAs have been shown to be involved in several pathological and physiological settings, including cardiovascular diseases and cardiac regeneration. In this Research Topic, the reports highlight the current knowledge and provide state-of-the-art data. In addition, obstacles and future challenges in ncRNA research and regenerative medicine are discussed.

## Author Contributions

JL and CB wrote and revised the manuscript. All authors contributed to the article and approved the submitted version.

## Funding

CB received funding from the Federal Ministry of Education and Research, Germany (research grant ERA-CVD JTC2018 INNOVATION, 01KL1903) and a Rebirth Synergy Grant from Förderung aus Mitteln des Niedersächsischen Vorab.

## Conflict of Interest

The authors declare that the research was conducted in the absence of any commercial or financial relationships that could be construed as a potential conflict of interest.

## Publisher's Note

All claims expressed in this article are solely those of the authors and do not necessarily represent those of their affiliated organizations, or those of the publisher, the editors and the reviewers. Any product that may be evaluated in this article, or claim that may be made by its manufacturer, is not guaranteed or endorsed by the publisher.
